# Co-Education

**Published:** 1911-11-25

**Authors:** 


### CO-EDUCATION.

The subject of co-education has recently been engaging the attention of students of education in this country, especially the co-education of the sexes during the years of adolescence. At a recent meeting of the Child Study Society, Dr. Kingsford, who lectured on the subject, was, on the whole, opposed to it, while Mr. Grant, who is the head of a co-educational school at Harpenden, and Miss Woods, of the Maria Grey Training College, as heartily supported it, and Mrs. Scharlieb, M.D., who presided, gave her testimony that the co-education of men and women medical students does no harm to the women, and, in the opinion of the principal of the college where she herself studied, has a beneficial influence on the men. These, of course, were men and women with a serious purpose in life.

Co-education is a subject on which it is not safe to dogmatise. It has the merit of bringing up individuals who will in all probability pass their lives in the most intimate relations with the opposite sex, with a knowledge of that other sex's characteristics, both of strength and weakness. The husband who has never looked upon woman as an angel, the wife who has never looked on man as a hero, are less likely to find bitter disappointment in married life than if the habits and customs of their co-partner were strange to them. These ends can be achieved wherever there is a mixed family of boys and girls, especially if they attend day-schools. For, after all, there must be a certain differentiation of curriculum between boys and girls, in view of the different work which, in all probability, they will have to do in after-life. This differentiation does not occur in the earlier years of education—both boys and girls must learn to read and write and spell. Both need some knowledge of history, geography, and arithmetic. But with the later years of school life—roughly speaking, with the "'teens," the need for varying the course of study with a view to the future work of the sexes becomes apparent. Co-education then becomes more or less incomplete; there is no special intellectual advantage in it, and the question is one of its moral worth. Is there any advantage in having the two sexes together at a time when sexual feeling is beginning to develop, though perhaps unconsciously? It is very doubtful. At the age of puberty many girls suffer from a morbid self-consciousness which makes them incline to withdraw from the old comradeship which they may have had with boys in earlier days, and it is generally better to let them do so until they are of an age fully to realise the meaning of womanhood. In other cases where a girl in her 'teens becomes forward and flirty, such a withdrawal from boys' society is even more desirable. In a few years she may develop into a fine and good woman, but if she does she will remember with many a blush the silliness of her early girlhood. There are other dangers, psychological as well as physiological, which many educationists overlook and with which, indeed, we are none of us too familiar owing to the sentimental prudery that prevents us from discussing the various aspects of the sex question. Boys who have had girl companions in play as well as in work cannot understand why the old playmate is now so unwilling to share in the rougher sports, and may urge the girls to a strenuous exertion which is physically inadvisable. The years from fourteen to eighteen should not be a time of either great intellectual or physical strain for girls, and as these are years of steadily increasing vigour of mind and body on the part of the average boy, such competition between the two sexes as is inevitable in co-education does not seem to be desirable. One thing at least seems certain—that though boys and girls who have been taught together since they entered the infant-room may go on studying together during the years of adolescence without much harm, if without any special advantage, it would be most unwise to begin co-education at the age when the meaning of sex is dawning on the mind.

